# Risk factors for presbycusis in a socio-economic middle-class sample

**DOI:** 10.1016/S1808-8694(15)30492-4

**Published:** 2015-10-19

**Authors:** Cláudia Simônica de Sousa, Ney de Castro, Erkki Juhani Larsson, Ting Hui Ching

**Affiliations:** 1 Master's degree student of otorhinolaryngology, Medical School (FCM) of the Sta. Casa de S. Paulo. Instructor of the otorhinolaryngology discipline at UNISA; 2 Doctor in medicine; adjunct professor of the otorhinolaryngology discipline at the FCM of the Sta. Casa de SP; 3 Physician, medical coordinator of the Check-Up Unit, H. A. Oswaldo Cruz de SP; 4 Master's degree in statistics, USP; graduate statistician at the FCM of the Sta. Casa de SP. Otorhinolaryngology discipline, FCM of the Sta. Casa de S. Paulo, Hospital Alemão Oswaldo Cruz de S. Paulo

**Keywords:** risk factors, sensorineural hearing loss, presbycusis

## Abstract

Presbycusis, or the aging ear, involves mainly the inner ear and the cochlear nerve, causing sensorineural hearing loss. Risk factors include systemic diseases and poor habits that cause inner ear damage and lead to presbycusis. Correct identification of these risk factors is relevant for prevention.

**Aim:**

To evaluate the prevalence and to identify the risk factors of presbycusis in a sample aged over 40 years. Study design: a retrospective case series.

**Subjects and Methods:**

medical records of 625 patients were evaluated. Presbycusis was identified using pure tone audiometry, speech audiometry and impedance testing of all patients.

**Results:**

The prevalence of presbycusis was 36.1%; the mean age was 50.5 years ranging from 40 to 86 years; 85.5% were male and 14.5% werf female. Age, the male gender, diabetes mellitus, and hereditary hearing loss were identified as risk factors. Cardiovascular diseases, smoking and consumption of alcohol were not confirmed as risk factors, although these have often been mentioned as risk factors for presbycusis.

**Conclusion:**

Notwithstanding the idea that presbycusis has multiple risk factors, this study identified few risk factors for this disease.

## INTRODUCTION

Presbycusis (from the Greek prébys = aged, and ákousis = hearing) refers to age-related hearing loss^1^ with no apparent cause. The term elderly or aged refers to persons aged 60 years or above, as defined by the World Health Organization (WHO) in 1984.^2^ The Brazilian Geographical and Statistical Institute (Instituto Brasileiro de Geografia e Estatística or IBGE) defines a middle social and economical class as that with a mean monthly income of seven to 15 Brazilian minimum salaries.^3^

According to the IBGE, the number of people aged over 60 years went from three million in 1960 to seven million in 1975 and to 14 million in 2002; this is a 500% increase over a 40-year period. Each year, an extra 650 thousand elderly people are added to the Brazilian population.^2^,^4^ Projections for 2024 suggest that there will be 32 million elderly persons in Brazil, about 15% of the population; this places Brazil in sixth place in the world classification of countries with the largest populations of elderly persons.^4^ There have been few epidemiological studies on the prevalence of presbycusis in Brazil.^5^,^6^

Presbycusis is characterized histopathologically by lesions in the inner ear and cochlear nerve, and functionally by sensorineural hearing loss.^7^,^8^ It starts in the fifth decade of life and is currently considered as the most frequent cause of adult hearing loss;^1^,^9^ thus, the onset of auditory system degeneration starts before the beginning of elderly age. The resulting communication difficulties, social withdrawal, depression and decreased quality of life cause significant health issues, and weigh significantly on the public health system.^2^

Risk factors such as systemic disease and poor habits add to presbycusis. According to the literature, risk factors are the male gender, smoking, noise exposure during leisure, stress, metabolic and vascular conditions (diabetes, dyslipidemias, systemic arterial hypertension, atherosclerosis, low encephalic output) and heredity.^5^, ^7^ The association between presbycusis and these risk factors, however, remains controversial and has not been well established. Nevertheless, these factors should be noted to eventually attenuate or prevent the effects of presbycusis.

## OBJECTIVE

The purpose of this study was to assess the prevalence of presbycusis and to correlate possible risk factors in a population sample aged 40 years and above, with or with no complaints of hearing loss.

## SERIES AND METHOD

An observational cross-sectional study with a sequential sample was undertaken of data gathered from the files of patients of both sexes aged 40 years and above at a check-up unit of Hospital X from January 2001 to August 2005. The Research Ethics Committee of the institution approved the study (protocol number 16/03 of 08/12/2003).

Patients undergoing check-up exams and evaluations at this unit generally have a university undergraduate degree and work in medium-sized and major companies; their income places them among the Brazilian middle to high social/economic class.

Based on inclusion and exclusion criteria, 625 files were selected.

### Inclusion criteria:

1. Subjects aged 40 years or over.
2. No vestibular complaints and no history of otological surgery.
3. Normal hearing characterized as:
  a. Pure tone thresholds (air conduction) over 25 dBHL at 250 Hz to 8 kHz
  b. Type A (Jerger) tympanometric curve.
4. Sensorineural hearing loss with a diagnosis of presbycusis according to:
  a. Bilateral and symmetrical hearing loss.
  b. Pure tone thresholds decreasing beyond 26 dBHL at least at 2 kHz to 8 kHz (flat or descending).10
  c. Type A (Jerger) tympanometric curve.

### Exclusion criteria:

1. Outer and/or middle ear disease.
2. Sensorineural hearing loss, with a clear cause other than presbycusis.
3. Patients working in noisy environments without adequate auditory protection.

A form generated by the main researcher established the data to be gathered from the files. The risk factors that were investigated were: sex, age, profession, diabetes mellitus, dyslipidemias (cholesterol and triglyceride levels), arterial hypertension, long-term use of medication, use of hormones, alcohol consumption (two or more times a week), smoking, a genetic history of age-related or idiopathic hearing loss.

Patients went through a check-up routine including otorhinolaryngological and audiological evaluations, laboratory exams, and clinical assessments of other medical specialties. The otorhinolaryngological exam (ENT) consisted of a physical examination and an audiological investigation. The same professionals (two physicians and two speech therapists) carried out the exams.

Speech therapists conducted standard voice and pure tone audiometry and immittance testing in an acoustic booth.^11^ An Interacoustics AC5 audiometer with TDH 19 headphones was used for voice and pure tone audiometry; an Interacoustics AZ7 device was used for acoustic immittance testing. These devices are calibrated annually.

Audiometry results were classified as normal or presbyacustic, regardless of the degree of hearing loss. Bivariate statistical analysis was used for testing the variables, evaluating the relation between presbycusis and possible associated risk factors.

Age was divided into six age groups: 40 to 45 years, 46 to 50 years, 51 to 55 years, 56 to 60 years, 61 to 65 years, and over age 65 years.

Two groups of professionals were established: engineers and others. Most of the sample consisted of engineers, which explained this division; there were a few other professions represented.

The chi-square statistical test was done to study the association among qualitative variables; the results of audiometric tests (outcome) and the Kruskal-Wallis non-parametric test were used for comparing the distribution of quantitative variables in altered audiometric tests. The significance level (p) was 5%.

The linear logistical regression model (multivariate analysis) was used for selecting those variables that best explained hearing loss. Variables with p < 0.20 in the bivariate analysis were used in the initial model. The backward method (Wald) was used for variable selection. The adjusted odds ratio (OR) of variables comprising the final model and their 95% confidence intervals (CI) were calculated.

The SPSS for Windows software, version 13.0, was used in this study.

## RESULTS

Files of male patients comprised 534 (85.4%) of 625 files. Presbycusis was found in 226 (36.1%) of these cases. The mean age in the sample was 50.5 years (SD – 6.7 years); the youngest patient was aged 40 years and the eldest, 86 years. Table 1 shows the social and demographic features of the sample and the distribution according to the chi-square test. The variables sex and age range were statistically significant (p < 0.05); the variable profession was not statistically associated with Presbycusis.

**Table 1:** Social and demographic features of the sample and the audiometric profile.

Variable	Audiometric Profile					
		Normal		Presbycusis		p*
		N	%	N	%	
Sex	male	327	82	207	91,6	0,001*
	female	72	18	19	8,4	
	40-45	92	23,1	22	9,7	0*
	46-50	131	32,8	47	20,8	
Age group	51-55	119	29,8	67	67	
	56-60	46	11,5	56	24,8	
	61-65	6	1,5	17	7,5	
	over 65	5	1,3	17	7,5	
Profession	Engineer	130	32,6	71	31,4	0,764
	Others	269	67,4	155	68,6	
Sub-total	399		226			

Table 2 shows the relation between clinical data and Presbycusis; the chi-square test revealed a positive statistical significance for the variables diabetes mellitus and a family history of hearing loss. There were non-significant associations with the variables: consumption of alcohol, smoking, a family history of dyslipidemia, systemic arterial hypertension, use of hormones, use of medication continuously, and other diseases.

**Table 2:** Results of clinical data and the audiometric profile.

Variable	Audiometric Profile						
		%	Normal		Presbycusis		p*
		N	%	N	%		
Family history of HL	No/Unknown	85,1	359	90%	173	76,50%	0*
	Yes	14,9	40	10%	53	23,50%	
SAH	No	66,7	275	68,90%	142	62,80%	0,121
	Yes	33,3	124	31,10%	84	37,20%	
DM	No	95,7	388	97,20%	210	92,90%	0,011*
	Yes	4,3	11	2,80%	16	7,10%	
Continuous use of medication	No	27,7	297	74,40%	155	68,60%	0,116
	Yes	72,3	71	31,40%	102	25,60%	
Use of hormone	No	92,2	368	92%	208	92%	0,93
	Yes	7,8	31	7,80%	18	8,00%	
Family history of Dyslipidemia	No/Unknown	90,6	361	90,50%	205	90,70%	0,924
	Yes	9,4	38	9,50%	21	9,30%	
Other Diseases	No	77,3	316	79,20%	167	73,90%	0,128
	Yes	22,7	83	20,80%	59	26,10%	
Smoking	No	77,9	313	78,40%	174	77%	0,674
	Yes	22,1	86	21,60%	52	23%	
Drinking alcohol	No	25,3	107	26,80%	51	22,60%	0,24
	Yes	74,7	292	73,20%	175	77,40%	
Sub-total			399		226		

p* significance level of the chi-square test.

There was no statistically positive association between the lipid profile and Presbycusis (Table 3).

**Table 3:** Results of the lipid profile and the audiometric profile.

Variable	Audiometric Profile			p*
	Normal	Altered	Total	
TG	345	215	560	0,086
Total cholestero	345	214	559	0,623
LDL - cholesterol	337	208	545	0,966

p* significance level of the Kruskal-Wallis non-parametric test.

The variables selected for the logistics regression model were those associated with Presbycusis in this sample. Table 4 shows that the female sex is a protective factor against hearing loss (OR < 1). Subjects aged over 50 years had a higher risk of Presbycusis, as did subjects with diabetes mellitus and a positive family history of hearing loss.

**Table 4:** Variables selected in the logistics regression model and the results.

Variable		p*	OR**	95,0%CI***	
				Lower	Higher
Sex	Female	0,003*	0,286	0,22	0,727
Age group	40-45	reference			
	46-50	0,321	1,367	0,738	2,535
	51-55	0*	1,953	1,07	3,564
	56-60	0*	3,677	1,908	7,089
	61-65	0*	10,851	3,441	34,213
	over 65	0*	12,217	3,875	38,519
Family history of HL	Yes	0*	3,098	1,853	5,18
DM	Yes	0,93	2,12	0,883	5,088

p* significance level of the backward method (Wald).

OR** odds ratio <1.

CI*** confidence interval.

Table 5 shows the results of the logistics regression model. A comparison between the prediction of the model and the audiometric profile in the sample revealed that the correctness percentage for patients having normal audiometric profiles was 80.3%, and 50.7% for patients having altered audiometric profiles. The total correctness percentage was 68.9%.

**Table 5:** Sensitivity and specificity of the inferred model.

Audiometric profile	Normal	Presbycusis	% correctness
Normal	277	68	80,3
Presbycusis	106	109	50,7
% Correctness			68.9

## DISCUSSION

According to histopathological studies, the onset of Presbycusis occurs in the fifth decade of life.^7^,^8^ The WHO has defined elderly as persons reaching or surpassing the seventh decade of life.^2^ The purpose of this study was to assess the risk factors of Presbycusis; thus, the age groups were established based on histopathological studies of the aging auditory system,^7^,^8^ comprising subjects from the fifth decade onwards.

Presbycusis is a multifactor process in which there is wide individual variation in the expression of each factor; the literature agrees on this fact.

The prevalence of Presbycusis was 36.1% in the sample. Published hearing loss prevalence values have ranged from 5% to 71.8%;^5^,^10^ population based epidemiological studies have indicated prevalence values from 20 to 40%.^13^,^14^ This variation in the literature is due mostly to variations in the method for detecting Presbycusis, which ranged from simple questionnaires to full audiological assessments.

Family income^15^ and the education level may be inversely proportional to the prevalence of Presbycusis. In the Brazilian study that presented a 71.8% prevalence - the highest found in our review - the sample consisted of a low income (one to two minimum salaries – 44.7% of the sample) and poorly educated population that was at a higher risk for poor health.^5^ Our results suggest that a sample of better educated and wealthier subjects - which have more access to information and health services - may control more adequately the risk factors for Presbycusis.

The method comprised a sample aged from the fifth decade onwards; according to the literature this is the age range in which Presbycusis occurs. The correlation between increasing age and a growing prevalence of Presbycusis was statistically significant, and conforms with published data.^16^, ^17^, ^18^

The sample consisted mostly of male subjects (85.4%), due to its demographic features: professionals in managerial jobs or directors of mid-sized and large companies; there are fewer women in these functions. The male gender was associated with Presbycusis in our study; this conclusion is reflected in other citations, wherein male subjects are at a higher risk for developing Presbycusis at a younger age.^6^,^14^ 17, 18, 19 This statement is, however, still an open issue; some references in the literature have suggested that the female gender is a risk factor for Presbycusis.^5^,^18^ Two papers concluded that there was not association between gender and Presbycusis.^17^,^20^

The percentage of engineers in our sample (47.40%) stimulated an investigation of this profession as a risk factor for Presbycusis, given the features of this activity. Subjects may be exposed to unhealthy environments from an auditory standpoint, which could lead to Presbycusis. There was, however, no positive association between engineering and Presbycusis. The explanation may be that most of the engineers in this sample were exposed to noisy environments for short periods and routinely used individual protection equipment (earplugs and/or earmuffs). A positive correlation between hearing loss and certain professions is frequent, although not necessarily characterized as noise-induced hearing loss.^14^,^21^

Findings in this study suggest a positive association between diabetes mellitus and Presbycusis; the type of diabetes was not specified. This association is well-known and generally accepted;^15^,^22^ some researchers, however, have question this correlation.^5^,^21^ Hearing loss in some forms of type I diabetes mellitus results from the inherited mitochondrial phenotype plus the effect of dysglycemia on the inner ear.^23^

Systemic arterial hypertension is often associated with Presbycusis,^12^,^24^ although a consensus is lacking since many studies have not established this correlation;^5^, ^15^, ^21^ this may be due to medical treatment of this condition. We found no association between systemic arterial hypertension and Presbycusis.

We also found no positive association between dyslipidemia and Presbycusis, a common finding in the literature.^24^, ^25^, ^26^ Some authors, however, have suggested that this association depends on the severity and duration of dyslipidemia.^12^,^27^

Hormone replacement therapy (progesterone and/or estrogen) has been suggested as a risk factor for Presbycusis.^28^, ^29^ We found no relation between Presbycusis and hormone replacement therapy, which might be due to the size of the sample.

Our results revealed no correlation between smoking/alcohol consumption and Presbycusis. These habits were coded qualitatively as present or absent in our questionnaire; there was no information about the duration and intensity of these habits. Some papers have raised the possibility of an association between smoking and Presbycusis,^30^, ^31^, ^32^ while others have not confirmed this correlation.^5^, ^15^, ^24^

There was a positive association between Presbycusis and a family history of this condition, which is a consensus in the literature. Genome studies have found X-linked probably mitochondrial genic loci affecting male subjects.^33^,^34^

## CONCLUSION

A study of risk factors for Presbycusis in social/economic middle-class subjects revealed that:

1. The prevalence of Presbycusis was 36.1%.
2. There were statistically positive associations between Presbycusis and: age, the male gender, diabetes mellitus and a family history of Presbycusis.
3. There was not association between Presbycusis and: profession, systemic arterial hypertension, dyslipidemia (triglycerides, total cholesterol, and low-density lipoprotein), a family history of dyslipidemia, and smoking or drinking alcoholic beverages.
4. Results show that although there are multiple risk factors for Presbycusis listed in the literature, these factors were limited in our sample.
